# Label-guided seed-chain-extend alignment on annotated De Bruijn graphs

**DOI:** 10.1093/bioinformatics/btae226

**Published:** 2024-06-28

**Authors:** Harun Mustafa, Mikhail Karasikov, Nika Mansouri Ghiasi, Gunnar Rätsch, André Kahles

**Affiliations:** Department of Computer Science, ETH Zurich, Zurich, 8092, Switzerland; Biomedical Informatics Group, University Hospital Zurich, Zurich, 8091, Switzerland; Biomedical Informatics, Swiss Institute of Bioinformatics, Zurich, 8092, Switzerland; Department of Computer Science, ETH Zurich, Zurich, 8092, Switzerland; Biomedical Informatics Group, University Hospital Zurich, Zurich, 8091, Switzerland; Biomedical Informatics, Swiss Institute of Bioinformatics, Zurich, 8092, Switzerland; Department of Information Technology and Electrical Engineering, ETH Zurich, Zurich, 8092, Switzerland; Department of Computer Science, ETH Zurich, Zurich, 8092, Switzerland; Biomedical Informatics Group, University Hospital Zurich, Zurich, 8091, Switzerland; Biomedical Informatics, Swiss Institute of Bioinformatics, Zurich, 8092, Switzerland; ETH AI Center, Zurich, 8092, Switzerland; Department of Biology, ETH Zurich, Zurich, 8093, Switzerland; The LOOP Zurich—Medical Research Center, Zurich, 8044, Switzerland; Department of Computer Science, ETH Zurich, Zurich, 8092, Switzerland; Biomedical Informatics Group, University Hospital Zurich, Zurich, 8091, Switzerland; Biomedical Informatics, Swiss Institute of Bioinformatics, Zurich, 8092, Switzerland; The LOOP Zurich—Medical Research Center, Zurich, 8044, Switzerland

## Abstract

**Motivation:**

Exponential growth in sequencing databases has motivated scalable De Bruijn graph-based (DBG) indexing for searching these data, using annotations to label nodes with sample IDs. Low-depth sequencing samples correspond to fragmented subgraphs, complicating finding the long contiguous walks required for alignment queries. Aligners that target single-labelled subgraphs reduce alignment lengths due to fragmentation, leading to low recall for long reads. While some (e.g. label-free) aligners partially overcome fragmentation by combining information from multiple samples, biologically irrelevant combinations in such approaches can inflate the search space or reduce accuracy.

**Results:**

We introduce a new scoring model, ‘multi-label alignment’ (MLA), for annotated DBGs. MLA leverages two new operations: To promote biologically relevant sample combinations, ‘Label Change’ incorporates more informative global sample similarity into local scores. To improve connectivity, ‘Node Length Change’ dynamically adjusts the DBG node length during traversal. Our fast, approximate, yet accurate MLA implementation has two key steps: a single-label seed-chain-extend aligner (SCA) and a multi-label chainer (MLC). SCA uses a traditional scoring model adapting recent chaining improvements to assembly graphs and provides a curated pool of alignments. MLC extracts seed anchors from SCAs alignments, produces multi-label chains using MLA scoring, then finally forms multi-label alignments. We show via substantial improvements in taxonomic classification accuracy that MLA produces biologically relevant alignments, decreasing average weighted UniFrac errors by 63.1%–66.8% and covering 45.5%–47.4% (median) more long-read query characters than state-of-the-art aligners. MLAs runtimes are competitive with label-combining alignment and substantially faster than single-label alignment.

**Availability and implementation:**

The data, scripts, and instructions for generating our results are available at https://github.com/ratschlab/mla.

## 1 Introduction

Sequencing databases are growing exponentially in size (Katz *et al.* 2021). In recent years, ‘sequence graphs’, in particular ‘De Bruijn graphs’ (DBGs), have become increasingly prominent models for representing and indexing large collections of sequencing data (Marchet *et al.* 2021), enabling improvements in both the scale and the accuracy of many biological analysis tasks, including genotyping (Hickey *et al.* 2020, Sirén *et al.* 2021), variant calling (Colquhoun *et al.* 2021, Krannich *et al.* 2021), and sequence search (Karasikov *et al.* 2020, Sirén *et al.* 2021).

Established search methods are designed for databases of assembled genomes (Morgulis *et al.* 2006). However, a large fraction of sequencing data deposited in archives like the Sequence Read Archive (SRA) or the European Nucleotide Archive have not yet been assembled (Harrison *et al.* 2020). This is because assembly requires expensive compute and human labour resources, done by first representing the overlaps between reads as an ‘assembly graph’ (e.g. a DBG), then extensively cleaning the graph (often requiring manual intervention), and finally assembling contiguous sequences (contigs) by graph traversal (Bankevich *et al.* 2022). Since genome assembly strives for long, high-quality contigs (Rahman and Medvedev 2022), the cleaning may discard a significant amount of signal from the sample (Sirén 2017, Rhie *et al.* 2021) with no guarantee that there will be no misassemblies among the final contigs (Rahman and Medvedev 2022).

To avoid these signal reduction and misassembly issues, a common way to compress and index a collection of unassembled read sets for search queries is to first construct and only lightly clean an assembly graph for each read set, then merge the graphs into a ‘joint assembly graph’ (Pandey *et al.* 2018, Karasikov *et al.* 2020). For indexing diverse sequencing datasets, light cleaning is still crucial to reduce the accumulation of noise when indexing diverse collections containing hundreds of thousands of samples (Karasikov *et al.* 2020). DBG-based indexing tools encode metadata as ‘graph annotations’, a key-value store associating each node with one or more metadata tracks, such as sample ‘labels’ (Iqbal *et al.* 2012, Almodaresi *et al.* 2018, Garrison *et al.* 2018, Pandey *et al.* 2018, Turner *et al.* 2018, Holley and Melsted 2020, Karasikov *et al.* 2020, 2022, Marchet *et al.* 2020, Fan *et al.* 2024). Similar to these previous works, we use accession IDs as labels to associate nodes back to their original database entries.

A key search task on these indexes is ‘sequence-to-graph alignment’, a generalization of pairwise sequence-to-sequence alignment (i.e. computing the maximum similarity ‘score’ between a ‘query’ and a ‘target’ sequence). In this setting, the target sequences are the spellings of contiguous walks (Section 2.1) on the sequence graph (Lee *et al.* 2002, Dvorkina *et al.* 2020, Ivanov *et al.* 2020, 2022, Li *et al.* 2020, Rautiainen and Marschall 2020, Schulz *et al.* 2021, Chandra and Jain 2023a, Joudaki *et al.* 2023, Ma *et al.* 2023, Rajput *et al.* 2023). Many of these tools follow a three-step ‘seed-chain-extend’ search paradigm (Section 2.2). This involves extracting and ‘anchoring’ query ‘seeds’ to the graph, using a ‘colinear chaining’ algorithm to construct anchor chains, and then ‘extending’ the chains via a search in the graph to form alignments.

### 1.1 Challenges when aligning to annotated DBGs

A large proportion of read sets in the SRA are sequenced at low depth (The fungi SRA samples indexed by Karasikov *et al.* (2020) have a median (mean) *k*-mer multiplicity of 9 (10.5); Supplementary Fig. 2.), producing heavily fragmented assembly graphs (Rhie *et al.* 2021). For metagenomics samples in particular, the constituent organisms are often sequenced at or below 1× coverage (Danko *et al.* 2021). Although the light cleaning applied to assembly graphs ensures that exact seed matches can be found (Karasikov *et al.* 2020), the long contiguous walks required for high-scoring (i.e. high-precision) alignments are often not present because of high graph fragmentation. This results in low recall, particularly for long reads, because the short alignments that can be found are not reported to maintain search precision.

Current alignment approaches for sequence graphs have limited support for fragmented graphs. The first approach is ‘label-free’ alignment, which ignores annotations during alignment. When applied to an annotated DBG representing a diverse cohort, these tools (Limasset *et al.* 2016, Garrison *et al.* 2018, Heydari *et al.* 2018, Dvorkina *et al.* 2020, Ivanov *et al.* 2020, 2022, Rautiainen and Marschall 2020) can meander search through a large search space inflated by biologically irrelevant sample combinations (Sirén *et al.* 2021, Karasikov *et al.* 2022). For this reason, these tools primarily target single-species pangenomes that often satisfy the assumption that all walks are biologically relevant. If this assumption holds, then these methods can overcome fragmentation in an individual sample’s assembly graph by combining sequence information from multiple samples since such contiguous walks are present in the joint graph. A second approach aligns queries to walks where all nodes in the walk share one (single-label) or more (label-consistent) common label(s) (Luhmann *et al.* 2021, Schulz *et al.* 2021). These tools suffer from low recall on fragmented assembly graphs, and so, this property also applies to joint annotated graphs because sample combinations cannot compensate for fragmentation. However, these tools do not suffer from search space inflation because all walks are biologically relevant. This is why these tools are applied to high-quality contiguous graphs indexing reference genomes or high-depth sequencing samples (Luhmann *et al.* 2021, Schulz *et al.* 2021). A third, recently emerging intermediate approach is ‘haplotype-aware’ alignment, which either aligns in a label-free fashion and scores recombinations afterwards (Rosen *et al.* 2017) or combines samples to a restricted degree by penalizing each combination during alignment (Avila *et al.* 2022, Chandra and Jain 2023b). These alignment strategies do not consider similarity (hence, biological relevance) when scoring a sample change and have so far only been applied to single-species pangenomes.

A property shared by all of these approaches is that a discontinuity in an individual assembly graph that does not overlap with another assembly graph will propagate to the joint DBG, limiting the joint graph’s contiguity. This stems from the approaches’ shared definition of alignment: the alignment target must be a contiguous walk.

### 1.2 Contribution: label-guided sequence-to-DBG alignment

Our goal in this work is to develop an alignment approach that can produce long accurate alignments to collections of low-depth sequencing samples represented by fragmented annotated DBGs. To this end, we propose a new alignment strategy called ‘multi-label alignment’ (MLA) designed for annotated DBGs. The strategy extends alignment scoring models with two key new operations: (i) ‘Label Change’ (LC) and (ii) ‘Node Length Change’ (NC). The label change operation penalizes traversals that change from one sample to a dissimilar sample, thus enhancing local single-character similarity scores with more informative global similarity. The node length change operation dynamically adjusts the node length, thus using shorter length nodes as proxies for missing nodes to locally improve graph connectivity (i.e. reducing fragmentation).

Efficiently implementing these operations must overcome several computational challenges. First, efficient anchor chaining relies on having a small number of anchors per query (Jain *et al.* 2022), an assumption that is easily violated in a multi-label setting. Second, sequence-to-graph alignment decision problems (i.e. does there exist an alignment) that allow for DBG edits is shown to be NP-complete (Gibney *et al.* 2022), necessitating heuristics to prevent excessive use of node length change operations.

To address these challenges, we implement MLA in a fast, approximate, yet accurate two-step approach: The first step is a new single-label seed-chain-extend aligner for annotated DBGs called SCA (Fig. 1.1–3), meant to reduce the computational burden of multi-label chaining by first performing single-label chaining with a traditional scoring model and extending the top chains among all labels to provide a preliminary alignment pool. SCA, adapts recent improvements in chaining to a DBG setting. The second step is a multi-label chainer called MLC (Fig. 1.4) that incorporates our novel multi-label scoring operations into its chain scoring. It extracts anchors from the alignments provided by SCA, resulting in a much smaller curated anchor set on which we apply our more expensive operations. It then performs multi-label chaining on these anchors and stitches the multi-label chains into alignments using fragments from SCAs alignments.

**Figure 1.**
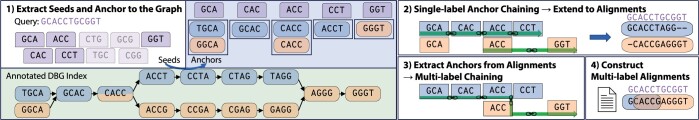
Computing multi-label alignments of a query sequence to an annotated De Bruijn graph. Each colour represents a label in the graph annotation, with some nodes (rounded boxes) having multiple labels. We first (1) extract seeds (half-rounded boxes, shaded seeds have no match) of length l≤k from the query sequence (in this example, k=4, l=3) and anchor the seeds to the graph, where each anchor (normal boxes) matches a seed to a node and a label (each bucket represents the node-label pairs to which a seed matches). Then, we (2) construct single-label chains (indicated by green lines) and extend them along single-label walks into alignments using SCA (note that a single-label walk does not exist from GGCA to CACC, so the yellow anchor GCA cannot be chained to ACC). We then (3) extract anchors from this alignment pool (reducing the anchor pool) and form multi-label chains using MLC, which allows label changes at matching *l*-mers. We (4) connect anchors using single-label alignment segments to form multi-label alignments.

**Figure 2.**
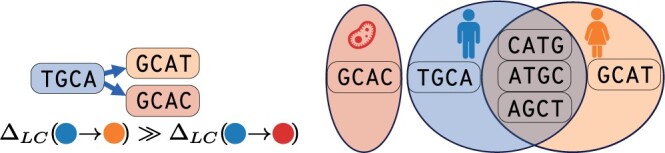
Label change scores measure sample similarity. $\Delta_{LC}$ measures the probability that a node with an orange label is also present in the blue sample but was not observed in that sample. We score a change to the orange label much higher because of the large overlap between the orange and blue *k*-mer sets.

**Figure 3.**
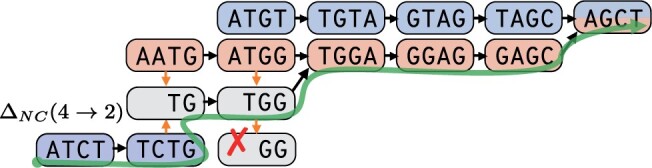
Traversal in a variable-order DBG overcomes graph disconnects to spell the sequence ATCTGGAGCT. Traversing from TCTG to its suffix TG allows for traversal to TGGA, so the red nodes compensate for the disconnect in the blue subgraph. In MLA, nodes of length l<k act as proxies for *k*-mers. In this graph, TGG stands in for CTGG. In our model, nodes of length <k may only traverse to longer nodes (e.g. TGG cannot traverse to GG). The green line represents the path taken to spell the sequence above and the orange arrows represent node length-changing traversals.

**Figure 4.**
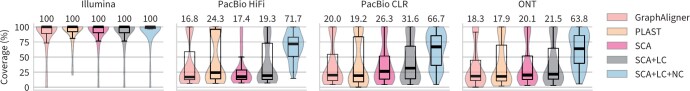
Coverage of each read’s best alignment. Coverage is the percentage of query characters covered by an alignment. SCA+LC uses our $\Delta_{LC}$, but not does allow node length changes. We report median coverages above each distribution.

Overall, we show in this work how our fast approximate implementation of MLA produces substantially longer and more accurate alignments compared to state-of-the-art aligners.

## 2 Preliminaries and background

### 2.1 Notation and definitions

A ‘string’ is a finite sequence of characters drawn from an alphabet Σ. $\Sigma^{k}$ denotes the set of all strings of length *k* (*k-mers*). For a string s=s[1]s[2]⋯s[l] of length |s|=l, with indices 1 ≤ i<j ≤ l, we denote a substring by s[i:j]:=s[i]⋯s[j]. The set of all *k*-mers extracted from a string set *S* is denoted by $K(S,k):=\underset{s\in S}{\cup }\overset{|s|-k+1}{\underset{i=1}{\cup }}\left\{s[i:i+k-1]\right\}$.

A ‘node-centric’ DBG of order *k* representing *S* has the nodes V:=K(S,k) and implicit edges $E(V):=\{(v_{1},v_{2})\in V^{2}:v_{1}[2:k]=v_{2}[1:k-1]\}$ (Chikhi and Rizk 2013). A ‘walk’ is a sequence of nodes $W=(v_{1},\ldots ,v_{m})$ where each $(v_{i},v_{i+1})\in E$. The ‘spelling’ of *W* is the string $T=v_{1}v_{2}[k]\cdots v_{m}[k]$. A ‘walk cover’ is a set of walks s.t. each node is visited by at least one walk. The ‘width’ of a graph is the number of walks in a minimal walk cover. An ‘annotated DBG’ has an auxiliary set of string labels L and an annotation $A:V\to 2^{L}$ associating each node with a label set. *W* is ‘label-consistent’ if $\overset{m}{\underset{i=1}{\cap }}A(v_{i})\neq \emptyset$. $V:L\to 2^{V}$ fetches all nodes with a given label.

We denote a query string by $Q\in \Sigma^{\left|Q\right|}$. A ‘sequence-to-graph alignment’ of *Q* to the ‘target string’ *T* along $W_{a}$ is a tuple $a=(Q_{a},T_{a},E_{a},W_{a})$, where $Q_{a}$ is a substring of *Q*, $T_{a}$ is a substring of *T*, and $E_{a}$ is a sequence of ‘edit operations’ transforming $T_{a}$ to $Q_{a}$. These operations are in {match, mismatch, insertion open, insertion extension, deletion open, deletion extension}. Each operation has a ‘score’, denoted by $\Delta_{=}$, $\Delta_{\neq }$, $\Delta_{IO}$, $\Delta_{IE}$, $\Delta_{DO}$, and $\Delta_{DE}$, respectively. Only $\Delta_{=}$ is positive, all other scores are negative. The score of *a*, denoted by $\Delta_{S}(a)$, is the sum of all edit operation scores, where a higher score indicates greater similarity. *a* is label-consistent if $W_{a}$ is label-consistent. Given two alignments $a_{1},a_{2}$ with respective substrings $Q[i_{1}:i_{1}+l_{1}-1],Q[i_{2}:i_{2}+l_{2}-1]$ s.t. $i_{1}<i_{2}$, we define $O(a_{1},a_{2}):=\min\{l_{2},i_{1}+l_{1}-i_{2}\}$. $a_{1}$ and $a_{2}$ ‘overlap’ if $O(a_{1},a_{2})>0$ and are ‘disjoined’ by a ‘gap’ of length −$O(a_{1},a_{2})$ otherwise. For a gap length $l\in N^{+}$, we denote the scoring model’s ‘gap penalty’ by $\Delta_{G}(l)$. In this work, we assume affine scoring (e.g. $\Delta_{G}(l):=\Delta_{IO}+(l-1)\Delta_{IE}$ for an insertion).

### 2.2 Sequence-to-graph alignment with seed-extend-chain

Modern approximate aligners predominantly use the ‘seed-chain-extend’ paradigm involving (i) ‘seed anchoring’, (ii) ‘colinear chaining’, and (iii) ‘anchor extension’ (Shaw and Yu 2023). A ‘seed’ is a query substring. An ‘anchor’ is a tuple of a seed and a graph walk spelling a superstring of the seed, where each node in the walk contributes at least one character to the spelling. A ‘chain’ is a sequence of anchors s.t. any two consecutive anchors are in order in both the query and the target, meaning that there exists a walk connecting their nodes (Mäkinen *et al.* 2019). Some works determine a traversal distance between nodes on-the-fly by traversing local neighbourhoods around nodes (Dvorkina *et al.* 2020, Li *et al.* 2020). More efficient strategies require a decomposition of the graph, typically into subgraphs (Chang *et al.* 2020, Rautiainen and Marschall 2020) or a path/walk cover (Mäkinen *et al.* 2019, Chandra and Jain 2023a, Ma *et al.* 2023, Rajput *et al.* 2023). After chaining, anchor extension searches the graph forwards and backwards from the ends of each anchor to find high-scoring walks.

## 3 Methods

### 3.1 General alignment workflow

Given a query *Q*, we find and anchor seeds, compute label-consistent alignments using SCA, then chain these alignments together into multi-label alignments using MLC. First, given a user-set seed length l ≤ k, we extract *l*-mer seeds from *Q* (Fig. 1.1) and anchor them by fetching all graph nodes with matching *l*-length suffixes and their associated labels (Fig. 1.2). An anchor α(i,l,v,ℓ) is a tuple in A:=N×N×V×L anchoring the seed Q[i:i+l−1] to a node *v*, with an associated label ℓ∈A(v). Afterwards, we find the top-scoring single-label chains (Fig. 1.3, Section 3.4). We extend each chain into a single-label alignment by using global alignment to connect consecutive anchors and ends-free extension from the first and last anchor in the chain. Given the resulting alignment pool, we extract all *l*-mer anchors and compute multi-label chains (Fig. 1.4, Section 3.5), incorporating label change ($\Delta_{LC}$) and node length change operations ($\Delta_{NC}$). We construct multi-label alignments from the top chains using segments from the label-consistent alignments.

### 3.2 Deriving a scoring model for novel alignment operations

We now detail the scoring model for our new operations for sequence-to-graph alignment: $\Delta_{LC}$ and $\Delta_{NC}$. For all constants defined in this section, refer to Section 4.3 for the values we use in our experiments. We define and extend two probabilistic graphical models for sequence-to-sequence alignment: a ‘target model’ and a ‘null model’, based on the probabilistic models presented by Frith (2019). Briefly, a model represents all possible mutations of an underlying target sequence, where a walk in a model emits edit operations that generate a query sequence (detailed in Supplementary Section S2.2). The score of any edit operation is the log  probability ratio of the operation’s corresponding transition probabilities in the target and the null model. The alignment score is the sum of these log  ratios (i.e. the query’s log-likelihood ratio). These sequence-to-sequence alignment models trivially induce analogous models for sequence-to-graph alignment by treating the spelling of each walk in a sequence graph as a separate target sequence and, hence, a pair of target and null graphical models.

#### 3.2.1 Deriving and computing label-change scores

For simplicity, suppose that we traverse along an edge $\left(v_{1},v_{2}\right)$ s.t. $A(v_{1})=\{\ell_{1}\}$ and $A(v_{2})=\{\ell_{2}\}$, where $\ell_{1}\neq \ell_{2}$. We denote this ‘label change’ from $\ell_{1}$ to $\ell_{2}$ as $\ell_{1}\to \ell_{2}$ and score this event using the probability that $v_{2}$ has label $\ell_{1}$ conditioned on $v_{2}$ having the label $\ell_{2}$. Intuitively, this is the probability that the *k*-mer $v_{2}$ observed in the sample with label $\ell_{2}$ is also present in the sample with label $\ell_{1}$, but was not observed (Fig. 2). To formulate this precisely, we extend our alignment models so that each graph-traversing operation emits a label change with transition probability $\mathrm{Pr}(\ell_{1}\to \ell_{2})$. Thus, the ‘label-change score’ is:

(1) $$\Delta_{LC}(\ell_{1}\to \ell_{2}):=\lambda_{LC}\left\lfloor {\;\text{log}\;}_{2}\frac{\mathrm{Pr}(\ell_{1}\to \ell_{2})}{{\mathrm{Pr}}_{0}(\ell_{1}\to \ell_{2})}\right\rfloor ,$$

where $\lambda_{LC}$ is a user-set scaling constant. For the null model, we assume no relationship between $\ell_{1}$ and $\ell_{2}$, so ${\mathrm{Pr}}_{0}(\ell_{1}\to \ell_{2}):=\mathrm{Pr}(\ell_{2})$, where Pr(ℓ) is the empirical probability of a label ℓ: $\mathrm{Pr}(\ell ):=\frac{\left|V(\ell )\right|}{\left|V\right|}$. For this new model to be reducible to a label-free setting, we require that $\Delta_{LC}(\ell \to \ell )=0$ (i.e. only score if the label changes) and that $\Delta_{LC}(\ell_{1}\to \ell_{2})\;\leq \;0$ (i.e. no label change increases the score). We satisfy these requirements if $\mathrm{Pr}(\ell_{1}\to \ell_{2}):=\mathrm{Pr}(v\in V(\ell_{1})\cap V(\ell_{2}))$, simplifying Equation (1) to:

(2) $$\Delta_{LC}(\ell_{1}\to \ell_{2})=\lambda_{LC}\left\lfloor {\;\text{log}\;}_{2}\mathrm{Pr}(\ell_{1}|\ell_{2})\right\rfloor .$$

Due to the log  in the definition of $\Delta_{LC}$ and the use of integers for alignment scores, we only require order-of-magnitude accuracy when computing $\mathrm{Pr}(\ell_{1}|\ell_{2})$. So, we approximate $\mathrm{Pr}(\ell_{1}|\ell_{2})$ using HyperLogLog++ counters (Heule *et al.* 2013) of $V(\ell_{1})$ and $V(\ell_{2})$, respectively, and the precomputed values $\left|V(\ell_{1})\right|$ and $\left|V(\ell_{2})\right|$. We use a false-positive probability of 0.05 for the counters and compute

(3) $$\mathrm{Pr}\left(\ell_{1}|\ell_{2}\right)\approx \frac{|V(\ell_{1})|+|V(\ell_{2})|-\left|\mathrm{HLL}(V(\ell_{1}))\cup \mathrm{HLL}(V(\ell_{2}))\right|}{\left|V(\ell_{2})\right|}.$$

#### 3.2.2 Deriving penalties for node length changes

Since low sequencing depth can produce disconnected graphs, one way to compensate for this is to allow for node insertions into the graph during the search. In this section, we describe how we use dynamic changes of the underlying DBGs order *k* during alignment as a more tractable proxy for inserting nodes.

Although MLC only utilizes node length change operations during chaining, we nonetheless incorporate this operation into our scoring model to derive its scoring function. For this, we switch our graph from a DBG of fixed order *k* to a variable-order DBG of maximum order *k* (Boucher *et al.* 2015).

Given a node *v* of length *k* with spelling *s* and a suffix length 1 ≤ l<k, a node length change is the traversal from *v* to the node with spelling s[k−l+1:k]. In our model, nodes with length *l* are proxies for missing *k*-mers (Fig. 3). However, we need to define penalties for changing the node length to prevent degenerate cases, such as searches along walks where every node has a short length (e.g. 1 or 2) that exist for every sequence over the alphabet.

To avoid this case, and to ensure that the model reduces to standard sequence-to-graph alignment on fixed-*k* DBGs, our scoring model does not penalize traversals that increase the node length by 1 or maintain the node length at *k*. Otherwise, we penalize traversal from a node of length *k* to one of length 1 ≤ l<k with a score $\Delta_{NC}(k\to l)$ and disallow all other node length-changing traversals. Consolidating these rules,

(4) $$\begin{array}{c} \Delta_{NC}(l_{1}\to l_{2}):=\{\begin{array}{cc} 0 & \text{if}\;l_{1}=l_{2}=k\;\text{or}\;l_{1}+1=l_{2}\\ (k-l_{2})\Delta_{J} & \text{if}\;l_{1}=k\;\text{and}\;l_{2}<k\\ -\infty & \text{if}\;l_{1}<k\;\text{and}\;l_{1}+1\neq l_{2} \end{array}, \end{array}$$

where $\Delta_{J}<0$ is a user-set constant.

### 3.3 Local colinear chaining on assembly DBGs

We now describe the modular chaining algorithm used by both SCA and MLC. Given anchors sorted by increasing end position (i.e. i+l), we perform local chaining using Algorithm 1, based on the more practical alternative algorithm implementation by Jain *et al.* (2022) (We fall back to the chaining algorithm from minimap2 (Li, 2018) with affine gap scoring if there are more than |Q| anchors (Section 3.6).). Their algorithm minimizes a nonnegative chain cost rather than maximizing an integer score, so we use the equations by Eizenga and Paten (2022) to convert scores into costs when evaluating our termination condition. This ‘forward pass’ computes optimal chaining scores. We reconstruct chains by ‘backtracking’, ensuring that we incorporate each anchor into, at most, one chain.

The helper function connect:A×A→Z approximates the score of a global alignment connecting anchor $\alpha_{1}$ to $\alpha_{2}$, with different implementations for SCA and MLC. Note that this score does not include $\alpha_{1}$ since its score is already included in the score vector $\Delta_{S}$ when computing updates.

Algorithm 1 Computing local colinear chaining scores.

**Input:** A sequence of anchors $\left(\alpha (i_{1},l_{1},v_{1},\ell_{1}),\ldots ,\alpha (i_{n},l_{n},v_{n},\ell_{n})\right)$ s.t. $x<y\Rightarrow i_{x}+l_{x}\leq i_{y}+l_{y}$. An initial guess of the maximum distance between anchors $b\in N^{+}$ and a scaling factor $b_{2}>1$.

**Output:** $\Delta_{S}\in Z^{n}$ s.t. $\Delta_{S}[j]$ is the best chaining score from any subsequence of $\left(\alpha (i_{1},l_{1},v_{1},\ell_{1}),\ldots ,\alpha (i_{j},l_{j},v_{j},\ell_{j})\right)$ ending with $\alpha (i_{j},l_{j},v_{j},\ell_{j})$.

1: $\Delta_{S}\leftarrow [\Delta_{=}\cdot l_{1},\ldots ,\Delta_{=}\cdot l_{n}]$

2: **repeat**

3:   F←1

4:   **for**L←1 to *n* **do**

5:    $F\leftarrow \min\left\{j\in \{F,\ldots ,L\}:i_{L}+l_{L}-\left(i_{j}+l_{j}\right)\leq b\right\}$

6:    **for**j←F to *L* s.t. $i_{j}<i_{L}$ and $i_{j}+l_{j}<i_{L}+l_{L}$**do**

7:     $s\leftarrow \Delta_{S}[j]+\mathrm{connect}(\alpha (i_{j},l_{j},v_{j},\ell_{j}),\alpha (i_{L},l_{L},v_{L},\ell_{L}))$

8:     $\Delta_{S}[L]\leftarrow \max\left\{\Delta_{S}[L],s\right\}$

9:    **end for**

10:     **end for**

11:   $b_{\text{last}}\leftarrow b$

12:   $b\leftarrow b\cdot b_{2}$ ▹ Increase max. distance between anchors

13:   $s_{\text{best}}\leftarrow \max\left\{\Delta_{S}[1],\ldots ,\Delta_{S}[n]\right\}$ ▹ Find the best score.

14:   $c_{\text{best}}\leftarrow \left\lceil 2\left(|Q|-\frac{s_{\text{best}}}{\Delta_{=}}\right)\right\rceil$ ▹ Convert score into a cost.

15: **until**$c_{\text{best}}>b_{\text{last}}$

16: **return**$\Delta_{S}$

### 3.4 SCA: single-label seed-chain-extend alignment

SCA implements colinear chaining and anchor extension algorithms for label-consistent alignment. After seed anchoring, we merge the anchors into maximal unique matches (MUMs). We then group the anchors by label and perform separate forward passes of the chaining algorithm for each group. Afterwards, we select the top ρ|Q| chains (with ties) among all labels and backtrack to construct these chains. ρ is a user-set chain density (Section 4.3).

To compute the connection score between two anchors $\alpha_{1}$ and $\alpha_{2}$, we sum a match score for the additional characters introduced by $\alpha_{2}$ to a gap penalty based on the absolute difference between (i) the difference of $\alpha_{1}$ and $\alpha_{2}$’s seed end positions in the query, and (ii) the traversal distance between their nodes in the graph (Supplementary Algorithm S1). To quickly estimate a traversal distance between two nodes along a label-consistent walk, we use walk covers of the subgraphs representing each label. A traversal distance is known if there exists a walk from $\alpha_{1}$ to $\alpha_{2}$ in the cover.

For each chain, we connect consecutive anchors using global alignment, then extend the first anchor backwards and the last anchor forwards using ends-free extension. For global alignment, we use TCG-Aligner (Karasikov *et al.* 2022) to ensure that each connecting walk is represented by the cover. For ends-free extension, we modify TCG-Aligners extender to restrict traversal to label-consistent walks. To further reduce the number of extensions, we discard chains that overlap with already-completed alignments. The result is a pool of label-consistent alignments.

### 3.5 MLC: multi-label colinear chaining

Given a pool of label-consistent alignments, we extract anchors from these alignments and construct multi-label chains (Fig. 1.4). Unlike the colinear chaining method in SCA, we have access to global alignment scores for connecting any in-order pair of anchors extracted from the same label-consistent alignment. We leverage this to define an anchor connection score that ensures that MLCs chaining scores equal the final MLA scores.

One property of MLCs scoring is that, given a chain of anchors from the same alignment and label, we only need the anchors at the beginning and end of the chain to compute the chain score. So, as a preprocessing step, we discard any anchor $\alpha (i_{j},l_{j},v_{j},\ell_{j})$ if it is the only anchor at position $i_{j}$ and if another anchor $\alpha (i_{j+1},l_{j+1},v_{j+1},\ell_{j})$ exists from the same alignment.

After the forward pass, we reconstruct the highest scoring chain for each label from the pool of label-consistent alignments. We construct a multi-label alignment from each chain by connecting consecutive anchors using alignment segments from the pool.

#### 3.5.1 Multi-label anchor connection scoring

We now detail MLCs anchor connection scoring (Supplementary Algorithm S2). Suppose we have anchors $\alpha (i_{j},l_{j},v_{j},\ell_{j})$ and $\alpha (i_{L},l_{L},v_{L},\ell_{L})$ from alignments $a_{x}$ and $a_{y}$, respectively. We consider three cases for the update score: (i) extending along the same alignment (i.e. x=y), (ii) connecting disjoint alignments (i.e. $O(a_{x},a_{y})\;\leq \;0$), and (iii) connecting overlapping alignments (i.e. $O(a_{x},a_{y})>0$).

- If x=y, then the anchor connection score is the score of $a_{y}$’s segment up to the longer query substring ending at $i_{L}+l$ (i.e. $\Delta_{S}(a_{y}[:i_{L}+l])-\Delta_{S}(a_{y}[:i_{j}+l])$).
- If $a_{x}$ and $a_{y}$ cover disjoint regions of *Q*, then the connection score includes the sum of $a_{x}$’s remaining score, the score of $a_{y}$’s segment up until $i_{L}+l$, the label change score, and the gap penalty for inserting extra query characters:
  (5) $$\Delta_{S}(a_{x})-\Delta_{S}(a_{x}[:i_{j}+l])+\Delta_{S}(a_{y}[:i_{L}+l])\begin{array}{c} +\Delta_{LC}(\ell_{j}\to \ell_{L})+\Delta_{DO}+\Delta_{G}(-O(a_{x},a_{y})). \end{array}$$
  To differentiate this case from (iii), we insert a sentinel $ character into the target spelling with an incurred $\Delta_{DO}$ score.
- If $a_{x}$ and $a_{y}$ cover overlapping regions of *Q*, assume that the current alignment segment of $a_{x}$ up until $i_{j}+l$ is of length ≥k and that $\left|a_{y}[i_{L}:]\right|\geq k$ (meant to avoid ambiguity when spelling a walk). We also only consider overlaps of length <k−1 (i.e. one cannot traverse from $a_{x}$ to $a_{y}$ with a single step without modifying the graph) since we assume that longer overlaps would have been discovered otherwise via normal graph traversal during anchor extension. We use a two-step procedure to try to connect the two anchors. First, we try to find an intermediate anchor $\alpha (i_{j^{\prime }},l_{j^{\prime }},v_{j^{\prime }},\ell_{L})$ from $a_{y}$ s.t. $i_{j}=i_{j^{\prime }}$ and hop to that node. Then we continue the traversal along $a_{y}$ towards $v_{j^{\prime }}$ to complete the connection. If such a node exists, the connection score is
  (6) $$\Delta_{S}(a_{y}[:i_{L}+l])-\Delta_{S}(a_{y}[:i_{j}+l])+\Delta_{LC}(\ell_{j}\to \ell_{L})+\Delta_{NC}(k\to l)\cdot 1_{v_{j}\neq v_{j^{\prime }}}.$$

Using Fig. 3 as an example, one such intermediate anchor is the node AATG sharing a 2-mer suffix with the node TCTG.

Note that this score does not depend on the length of $v_{j}$ and $v_{j^{\prime }}$’s longest common suffix if $v_{j}\neq v_{j^{\prime }}$. We motivate this design choice with the following observation: If there is a single character mismatch between position $k-l^{\prime }+1$ (where $l^{\prime }$ need not equal the seed length *l*) in a node *v*’s spelling and position $k-l^{\prime }$ in another node $v^{\prime }$’s spelling that prevents the nodes from being adjacent (e.g. *v* spells GATGC and $v^{\prime }$ spells ACGCT for k=5 and $l^{\prime }=3$), then we require $l^{\prime }$ node insertions to create a path to $v^{\prime }$ from a predecessor of *v*, despite the ground truth that it originated from a single substitution (for our example, given appropriate characters c,d∈Σ, these new nodes would spell cdGAC, dGACG, and GACGC). Thus, defining the connection score as a function of $k-l^{\prime }$ instead of k−l would induce unequal scores for each value of $l^{\prime }$, even though all of these edit events are equally likely.

### 3.6 Time and space complexity

Suppose we have *n* sorted anchors (incurring a worst-case complexity of O(n log n) if the seeder does not produce sorted anchors). Let *C* denote the time to execute a connect function. If n ≤ |Q|, then Algorithm 1 has an average-case time complexity in $O(c^{*}|Q|C)$, where $c^{*}$ is the minimum chain cost (Jain *et al.* 2022). If n>|Q|, then we fall back to the chaining algorithm from minimap2 (Li 2018) with a worst-case time complexity in O(bnC) for the forward pass, where *b* is a user-set bandwidth parameter. Although we perform ρ|Q| backtracking procedures, since each anchor is incorporated into at most one chain, we terminate a procedure as soon as we reach an already-chained anchor. So, backtracking takes worst-case O(n) time. Alongside the *n* anchors, we store Θ(1) information per anchor for the chaining scores and backtracking information, so the total space complexity is in Θ(n).

Consider SCAs connect. Suppose that *Q* maps to *L* labels, with corresponding anchor counts $n_{1},\ldots ,n_{L}$, walk cover sizes $W_{1},\ldots ,W_{L}$, and optimal chaining costs $c_{1}^{*},\ldots ,c_{L}^{*}$. Since MUMs generally satisfy $n_{i}\;\leq \;|Q|$ (Jain *et al.* 2022), the average-case time complexity of SCAs forward passes is $O\left(|Q|\sum_{i=1}^{L}c_{i}^{*}W_{i}\right)$. After extension, we extract *m* anchors from the resulting alignments.

Considering MLC, it is clear that $C_{\text{MLA}}\in O(1)$. Since we chain anchors from multiple labels in a single forward pass, we can no longer assume that m ≤ |Q| will generally hold. Thus, multi-label chaining has an average-case time complexity in $O(c^{*}|Q|)$ when m ≤ |Q| and worst-case time complexity in O(bm) otherwise. Splicing alignment segments to convert a multi-label chain into an alignment that spells *T* runs in worst-case O(|T|) time.

## 4 Evaluation methodology

We compare our methods to GraphAligner (Rautiainen and Marschall 2020), a state-of-the-art tool for traditional sequence-to-graph alignment to DBGs, and PLAST (Schulz *et al.* 2021), a BLAST-like tool for label-consistent alignment to annotated DBGs. We implement two additional baselines: SCA+LC100 implements multi-label chaining with $\Delta_{LC}=-100$ (plotted in Supplementary Fig. S3), while SCA+LC implements our $\Delta_{LC}$ but disallows node length changes. Following this terminology, we denote our full MLA method of SCA+MLC as SCA+LC+NC. We evaluate all tools on a simulated joint assembly graph indexing 3042 fungi genomes from GenBank (Sayers *et al.* 2022).

### 4.1 Simulating assembly graphs and query sequences

From each genome, we simulate a 10×-coverage Illumina HiSeq-type read set using *ART* v2.5.8 (Huang *et al.* 2011) and construct a cleaned assembly DBG of order k=31 using the procedure in MetaGraph (Karasikov *et al.* 2020). Briefly, this involves pruning graph tips of length <2k and discarding any unitig whose median *k*-mer count is below a noise count threshold computed from the sample’s *k*-mer spectrum. We then merge these graphs into an annotated DBG with nodes labelled by their source accession IDs. To merge the graphs, we compute walk covers for each graph and construct the joint graph from these sequences.

We preprocess the graph and generate walks using the procedure described by Rajput *et al.* (2023). To reduce the final representation size, we refrain from maintaining a matrix of traversal distances from each node to each stored walk. Instead, we augment each cover with additional walks spanning the edges discarded during preprocessing. We losslessly encode the walks as coordinate graph annotations (Karasikov *et al.* 2022). We use the GFA representation of the joint graph to interface with GraphAligner and the walk covers to construct the PLAST index.

We simulate query reads from the same genomes with a different random seed, using *ART* for Illumina HiSeq-type reads and pbsim3 (Ono *et al.* 2022) for PacBio Sequel CLR-type, PacBio Sequel HiFi-type, and ONT-type reads. We generate HiFi reads from simulated 10-pass Sequel subreads using the PacBio ccs tool. We form a query set for each read type by drawing 100 random reads from the pool of reads aggregated from all genomes.

### 4.2 Recall-, coverage-, and taxonomy-based measures

Alongside read mapping quality, we measure the accuracy of the retrieved labels w.r.t. the ground-truth genomes using ‘taxonomic profiles’ since our query reads are simulated from known genomes.

Taxonomic classification accuracy is highly dependent on which alignments are reported in a read mapping. So, we first find an appropriate score cut-off to determine which alignments to report for each read. Since our test reads are of different lengths, we divide each score by the length of its corresponding query to get a ‘relative score’. Given the alignments of a read sorted by decreasing relative score and a cut-off, we select alignments by greedily picking a disjoint subset whose relative scores are above the cut-off. We vary the cut-off from 0.0 to 1.0 in steps of 0.02. For each cut-off, we evaluate the (i) recall and the (ii) mean taxonomic profile error. We measure taxonomic profile error using the WGSUniFrac error of the profile relative to the ground-truth profile (Wei and Koslicki 2022), a measure of the fraction of the taxonomic tree traversal distance that differs between the two profiles. For easier interpretation, we define the ‘UniFrac accuracy’ as 1.0−WGSUniFrac error. Since taxonomic IDs are not available for all strains, we generate a custom taxonomic tree by augmenting the NCBI Taxonomy with a new leaf node for each GenBank accession ID. The ground-truth profile of a read states that its ground-truth accession is 100% abundant, whereas the profiles computed from alignments weight each accession by the fraction of query characters covered by alignments to that accession.

### 4.3 Experimental setup and code availability

We performed all experiments on an AMD EPYC-Rome processor from ETH Zurich’s high-performance compute systems using a single thread and a seed size of l=19, with default parameters for all tools except for the following: For scoring, we set $\Delta_{=}=1$ and $\Delta_{\neq }=\Delta_{IO}=\Delta_{IE}=\Delta_{DO}=\Delta_{DE}=-1$. For SCA and MLC, we use a chain density of ρ=0.01 and chaining parameters b=400 and $b_{2}=4$. In MLC, we set the label-change score scaling factor to $\lambda_{LC}=\Delta_{=}$ and the node length change scaling factor to $\Delta_{J}=\Delta_{DE}$. We motivate these choices in Supplementary Section S2.4. We use the BOSS representation to index our DBGs, which natively supports the subset of variable-order DBG operations used for node length changes (Bowe *et al.* 2012). We implemented our methods within the GPLv3-licensed MetaGraph framework (Karasikov *et al.* 2020) hosted at https://github.com/ratschlab/metagraph.

## 5 Results and discussion

### 5.1 Assembly graphs are much wider than pangenomes

Our simulations produced read sets with a median (mean) of 700 470 (1 768 812) *k*-mers per sample. These resulted in graphs with a median (mean) size of 48 660 (122 826) *k*-mers (Supplementary Fig. S1). After merging these assembly graphs, the annotated DBG contains 187 662 586 *k*-mers and its index consumes 179 MB. We report execution statistics for graph indexing in Supplementary Section S2.5.

We observe that unlike the pangenome graphs explored in previous chaining works with widths ranging from 1 to 60 (Chandra and Jain 2023a, Ma *et al.* 2023, Rajput *et al.* 2023), our assembly graphs are much wider, with a median (mean) width of 221 (542.8). These observations corroborate our choice to refrain from encoding a full node-to-walk distance matrix for SCA.

### 5.2 Multi-label alignments are substantially longer, computed at competitive execution times

For all read types, we observe that SCA+LC+NC produces the longest alignments (Fig. 4), with median query coverage increases of 47.4% for HiFi, 46.7% for CLR, and 45.5% for ONT reads, respectively, relative to the next-best state-of-the-art aligner. All methods have median coverages of 100% for Illumina reads, with the smallest interquartile range observed for SCA+LC+NC. Our baselines SCA+LC100 and SCA+LC achieve similar or slightly improved performance relative to SCA, but far below SCA+LC+NC. PLAST has the highest third quartile coverage for PacBio reads among all tools and higher median coverage on HiFi reads than the most comparable tool SCA. However, its execution time is ∼280–1200× slower than SCA due to its default behaviour of extending a large number (200) of high-scoring matches per query (Fig. 7).

### 5.3 Multi-label alignment maintains the best classification accuracy at most read mapping recall levels

**Figure 5.**
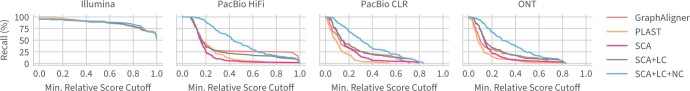
Read mapping recall for different relative score cut-offs. For each relative score (i.e. score scaled by query length) cut-off, the recall is the fraction of reads with an alignment scoring at least at that cut-off.

**Figure 6.**
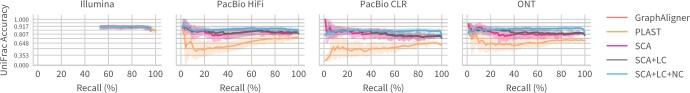
Mean UniFrac accuracy of all mapped reads at different read mapping recall levels. UniFrac accuracy measures the similarity between a read’s taxonomic profile (induced from the labels of all reported alignments) and its ground-truth profile, similar to precision. Shading indicates 95% confidence intervals of the mean estimated from 1000 bootstrap samples. Based on our interpretation of WGSUniFrac error values (detailed in Supplementary Section S2.3), each *y*-axis grid line corresponds to the midpoint value of a taxonomic rank (accession, strain, species, genus, etc.). The *y*-axis is scaled to better emphasize the upper range of UniFrac accuracy values, corresponding to accuracy at lower taxonomic ranks. We exclude GraphAligner since it does not consider labels.

**Figure 7.**
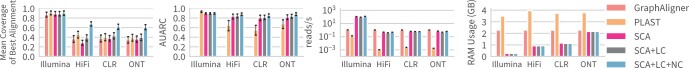
Comparison of performance measures for each alignment tool. AUARC is the area under the UniFrac Accuracy–Recall curve (Fig. 6), where higher values indicate greater accuracy. Error bars indicate 95% confidence intervals calculated from 1000 bootstrap samples. See Supplementary Table S3 for the numbers plotted here.

Sweeping through different relative score cut-offs, we observe that the full MLA (SCA+LC+NC) method has the greatest recall on error-prone reads for all cut-offs and the greatest recall for HiFi reads at cut-offs below 0.6 (Fig. 5). All tools perform similarly on Illumina reads. When comparing taxonomic profiles, SCA+LC+NC has the greatest UniFrac accuracy for long reads at recall values above 20% for HiFi and ONT reads and above 50% for CLR reads (Fig. 6). All tools perform similarly on Illumina reads. The areas under the mean UniFrac Accuracy-Recall curves (AUARCs) for Illumina reads are 0.930 for PLAST and 0.895 for our methods (Fig. 7). For all long-read types, SCA+LC+NC has the highest AUARCs, ranging from 0.852 to 0.882, compared to SCA (0.806–0.835), SCA+LC100 (0.811–0.844), SCA+LC (0.815–0.845), and PLAST (0.548–0.677). We infer that MLA improves accuracy by chaining together alignments to related samples.

### 5.4 Overall performance

Overall, multi-label alignments are substantially longer than alignments produced by other tools, while maintaining competitive execution times and the lowest RAM usage (Fig. 7). We decompose our methods’ execution times into their different components in Supplementary Table S4. For Illumina reads, SCA and SCA+LC+NC are substantially faster than all other tools. GraphAligner is the fastest tool for long reads, while SCA and SCA+LC+NC are comparable.

## 6 Conclusions

In this work, we presented the MLA strategy, a novel alignment scoring model that compensates for disconnects in low-depth assembly graphs by combining sequence information from multiple related samples and improving graph connectivity during alignment. We implement MLA on annotated DBGs with a two-step process: SCA computes label-consistent alignments and MLC computes multi-label alignments from these label-consistent alignments.

Since SCA does not encode a traversal distance matrix from each node to each walk encoded by the graph annotation, providing such a matrix can potentially increase label-consistent alignment lengths and consequently provide a stronger basis for MLC. Our observation that PLAST produces longer alignments than SCA on low-error reads provides further evidence that a more rich traversal distance index can improve SCAs alignment performance. Despite the large widths of assembly graphs, we expect this matrix to be sparse and, hence, easily compressible.

Another possible extension is merging SCA and MLC into a single holistic seed-chain-extend procedure. We maintained these two steps in this work to provide a small number of anchors to each chaining run. One can explore how well our current approach approximates this unified approach and how to approximate an ends-free extension incorporating label and node length changes. In this context, there are a few possibilities for implementing the node length change operation into chain scoring. These include using the current procedure of finding intermediate suffix-sharing nodes and a more sensitive, but daunting approach of representing walk covers for all desirable node length values l<k.

Although our algorithms are implemented within the MetaGraph framework, the concepts from our methods can readily be applied in other pangenome graph frameworks and potentially see more widespread use. These methods make unassembled read sets a more powerful resource for bioinformatics research.

## Supplementary Material

btae226_Supplementary_Data

## Data Availability

The data (including accession IDs for all reference genomes), scripts, and instructions for generating our results are available at https://github.com/ratschlab/mla.
