# Supplementary material for: Label-guided seed-chain-extend alignment on annotated De Bruijn graphs
Source: Bioinformatics. 2024 Jun 28;40(Suppl 1):i337–46. doi: 10.1093/bioinformatics/btae226 (PMC11211850; doi:10.1093/bioinformatics/btae226)
Supplement: btae226_Supplementary_Data [file btae226_supplementary_data.pdf]

1. Supplementary Algorithms

Algorithm 1 connect$(\cdot,\cdot)$ function used by `SCA` to calculate the score for connecting two anchors with the label ℓ.

Input: Anchors $\alpha(i_j, l_j, v_j, \ell_j)$ and $\alpha(i_L, l_L, v_L, \ell_L)$ with label ℓ such that $i_j < i_L$ and $i_j + l_j < i_L + l_L$. A walk cover $\mathcal{W} = \{(v_{11}, \ldots, v_{1n_1}), \ldots, (v_{w1}, \ldots, v_{wn_w})\}$ of the subgraph of all nodes with label ℓ. Match open score $\Delta_= > 0$. Gap penalty function Δ_G.

Output: A score $s \in \mathbb{Z}$.

$d_Q \leftarrow i_L + l_L - (i_j + l_j)$
$g_{\text{best}} \leftarrow \infty$
for all $(v_1, \ldots, v_n) \in \mathcal{W}$ **do**
  if $(v_j, v_L) = (v_x, v_y)$ and $x < y$ **then**
   $g_{\text{best}} \leftarrow \min\{g_{\text{best}}, |y - x - d_Q|\}$
  end if
end for
return $\Delta_= \cdot \min\{d_Q, l_L\} + \Delta_G(g_{\text{best}})$

Algorithm 2 connect$(\cdot,\cdot)$ function used by `MLC` to calculate the score for connecting two anchors of length l.

Input: Anchors $\alpha(i_j, l_j, v_j, \ell_j)$ and $\alpha(i_L, l_L, v_L, \ell_L)$ that originate from alignment a_x to label ℓ_j and a_y to label ℓ_L, respectively, such that $i_j < i_L$. A deletion open score $\Delta_{DO} < 0$. Gap penalty function Δ_G.

Output: A score $s \in \mathbb{Z}$.

if $x = y$ **then**
  if $\ell_j = \ell_L$ **then return** $\Delta_S(a_y[: i_L + l]) - \Delta_S(a_y[: i_j + l])$
  else return $-\infty$
  end if
end if
if current segment of a_x has length $< k$ or $|a_y[i_L :]| < k$ **then**
   return $-\infty$
end if
$s \leftarrow \Delta_{LC}(\ell_j \rightarrow \ell_L)$
$o \leftarrow O(a_x, a_y)$
if $o \leq 0$ **then**
   return $s + \Delta_{DO} + \Delta_G(-o) + \Delta_S(a_x) - \Delta_S(a_x[: i_j + l]) + \Delta_S(a_y[: i_L + l])$
end if
if $\alpha(i_{j'}, l_{j'}, v_{j'}, \ell_L)$ from a_y exists s.t. $i_{j'} = i_j$ **then**
  if $v_j \neq v_{j'}$ **then**
    $s \leftarrow s + \Delta_L(k \rightarrow l)$
  end if
   return $s + \Delta_S(a_y[: i_L + l]) - \Delta_S(a_y[: i_j + l])$
end if
return $-\infty$

2. Supplementary Sections

2.1. Merging single-label anchors into MUMs

We define a *unipath* as a maximal non-branching path in a DBG whose spelling is called a *unitig*. In the initial anchor set, all anchors are of the same length l. Then, given two consecutive anchors $\alpha(i_1, l_1, v_1)$ and $\alpha(i_2, l_2, v_2)$ (since we may be merging into a previously merged anchor), if $i_1 + l + 1 = i_2 + l_2$, if these are the only anchors with end positions $i_1 + l$ and $i_2 + l_2$, if $(v_1, v_2) \in E$, and if v_1 and v_2 are in the same unipath, we merge them into a single anchor of length $l_2 + 1$.

2.2. Probabilistic graphical alignment models

Given a starting node in the graph, the first generative model (called the *target model*) generates query sequences with probabilities proportional to their similarity to the spellings of walks that originate at the starting node, assuming that the query sequence is generated by a series of edits to a walk spelling. The second model (called the *null model*) generates both query sequences and graph walks with probabilities only proportional to the sequences' respective lengths, assuming that the two tasks occur independently. Each alignment operation E (defined in Main Section 2.1) corresponds to traversing an edge in the graphical model (called a *transition*), which occurs with a transition probability denoted by $\Pr(E)$ for the target model and $\Pr_0(E)$ for the null model. The graphical model *emits* a query character if $E \in \{=, \neq, IO, IE\}$ and it traverses a graph edge to emit a target character if $E \in \{=, \neq, DO, DE\}$. We define the score of E as

$$\Delta_E := \lambda_E \left\lfloor \log_2 \frac{\Pr(E)}{\Pr_0(E)} \right\rfloor, \tag{1}$$

where λ_E is a user-set scaling constant. Thus, the sum of all operation scores (i.e., the alignment score Δ_S) corresponds to the log-likelihood ratio of the corresponding walk probabilities in the target and null models.

2.3. Inferring taxonomic ranks from WGSUniFrac values

When computing WGSUniFrac errors, the algorithm assigns a branch length of x^{-1}, where x is the distance from the branch to the tree root. We consider nine different ranks: superkingdom, phylum, class, order, family, genus, species, strain, accession, with corresponding branch lengths $\left(\frac{1}{2}, \frac{1}{3}, \frac{1}{4}, \frac{1}{5}, \frac{1}{6}, \frac{1}{7}, \frac{1}{8}, \frac{1}{9}\right)$. If we assume that the taxonomic profile of a read contains a single accession (and by definition, the ground-truth profile has a single accession), then the unweighted WGSUniFrac error for accuracy at level x (meaning a mismatch at rank $x+1$) is $2 \cdot \sum_{i=x+1}^{9} \frac{1}{i}$ since there is an incorrect branch in both profiles. Since the maximum WGSUniFrac error value is $\sim$5.66, each unweighted error is divided by this constant. Based on this interpretation, we calculate midpoints for WGSUniFrac error and accuracy value ranges corresponding to classification accuracy at least taxonomic rank in Table 1.

Table 1. Midpoint WGSUniFrac error and accuracy values for matching accuracy at each taxonomic rank. UniFrac accuracy is $1 -$ WGSUniFrac error.

Taxonomic Rank	WGSUniFrac Error Midpoint	UniFrac Accuracy Midpoint
Accession	0.0	1.0
Strain	0.039	0.961
Species	0.083	0.917
Genus	0.134	0.866
Family	0.193	0.807
Order	0.264	0.736
Class	0.352	0.648
Phylum	0.470	0.530
Superkingdom	0.647	0.353
Root	1.0	0.0

2.4. Motivations for alignment parameters

Our value of $\Delta_J := \Delta_{DE}$ is motivated by the fact that we use node length changes as a proxy for inserting nodes into the graph. Thus, we score each unit change in node length with the same penalty as a character insertion into the reference (i.e., a deletion from the query). We choose a linear penalty model instead of affine because the probability of a false anchor chaining grows exponentially as the node length difference changes (unlike the argument used to support affine penalties in traditional alignment gap scoring that the length of an indel is less important than the event of an indel being opened).

The choice of the chain density parameter ρ is meant to provide a sufficiently rich number of single-label alignments to MLC while not incurring a high anchor extension cost (Tab. 2). For Illumina, HiFi, and CLR reads, a value of 0.01 leads to the greatest mean query coverage, while for ONT reads, the ρ values leading to the top two mean coverages come with a significant alignment time cost.

The chaining parameter $b_2 := 4$ is the same value used by the 2022 co-linear chaining work by Jain et al. (2022) on which our algorithm was based. Due to our anchor filtration in `MLC` (Main §3.5), we expect a larger distance between anchors since many anchors that occur in only one single-label alignment are considered to be redundant and discarded. Thus, we use $b := 400$ instead of 100 to avoid one iteration of the `while` loop in Main Algorithm 1.

Table 2. Parameter sweep of the chain density parameter ρ for MLA (SCA+LC+NC). Mean total coverage is the mean percentage of query characters that are covered by at least one alignment.

Platform	ρ	Alignment Time (s)	Mean Total Coverage (%)
Illumina	0.001000	0.835939	90.326700
	0.003000	0.806182	90.326700
	0.006000	0.820456	90.326700
	0.010000	0.815084	90.326700
	0.030000	0.817829	90.326700
	0.100000	1.032633	90.326700
PacBio HiFi	0.001000	125.503234	41.082400
	0.003000	154.885777	67.081900
	0.006000	188.011765	72.027600
	0.010000	223.664908	**74.175000**
	0.030000	319.640846	71.327200
	0.100000	515.824620	73.557600
PacBio CLR	0.001000	27.134141	44.229900
	0.003000	93.205197	64.263500
	0.006000	153.523381	69.614300
	0.010000	168.409920	**70.033700**
	0.030000	281.570056	68.621300
	0.100000	323.951430	69.366700
ONT	0.001000	36.809423	39.874400
	0.003000	88.980369	64.061200
	0.006000	143.971906	69.011700
	0.010000	170.929845	69.136300
	0.030000	400.434129	**69.794000**
	0.100000	444.226997	69.761300

2.5. Graph construction and indexing

For the following operations, we report times for execution on a single thread. Cleaning all input read files took 190m50.05s. From the cleaned sequence sets, assembling all walk covers took 46m58.23s. Constructing the BOSS representation of the joint graph from the walk covers took 2m58s. Afterwards, annotating the joint graph took 33m1.65s. Finally, constructing the HyperLogLog++ sketches of the annotation took 8.16s. We note that the input cleaning, walk cover construction, and annotation of the joint graph can be run concurrently in a distributed fashion on multiple compute nodes. Joint graph construction can run with multiple threads. Converting the graph to the GFA format for `GraphAligner` took 2m50s. Construction of the `PLAST` index from the walk covers took 19m47s.

3. Supplementary Figures

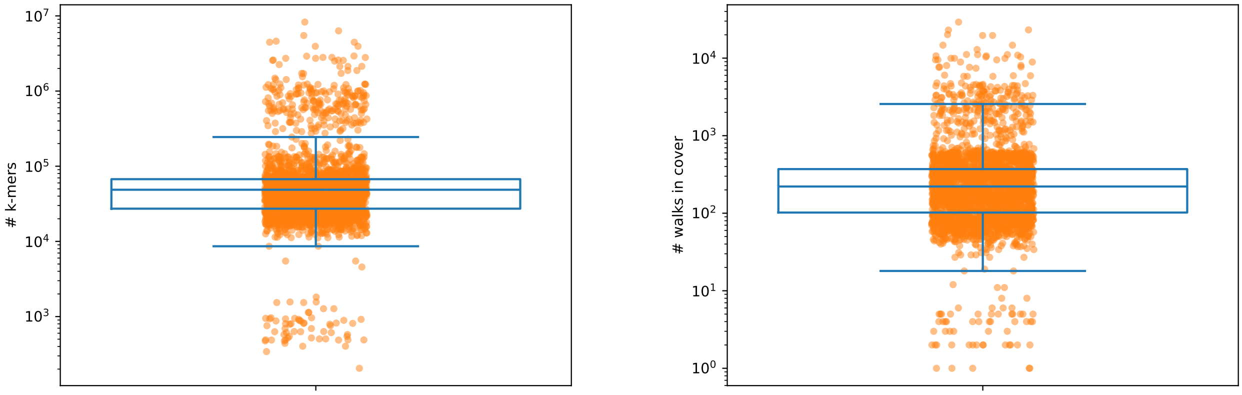

Fig. 1: **Number of k-mers (left) and walk cover sizes (right) in the simulated assembly graphs.**.

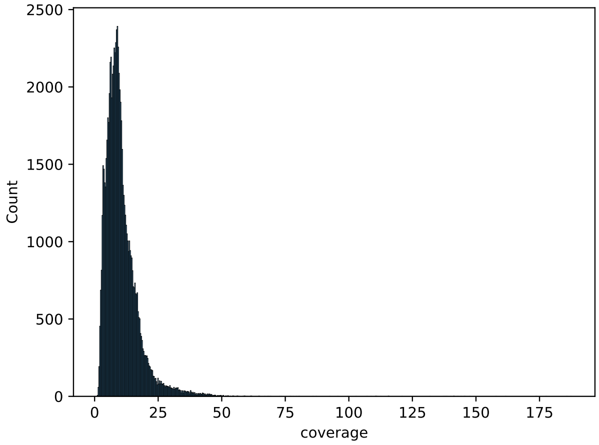

Fig. 2: **k-mer count distribution of SRA Fungi samples.**

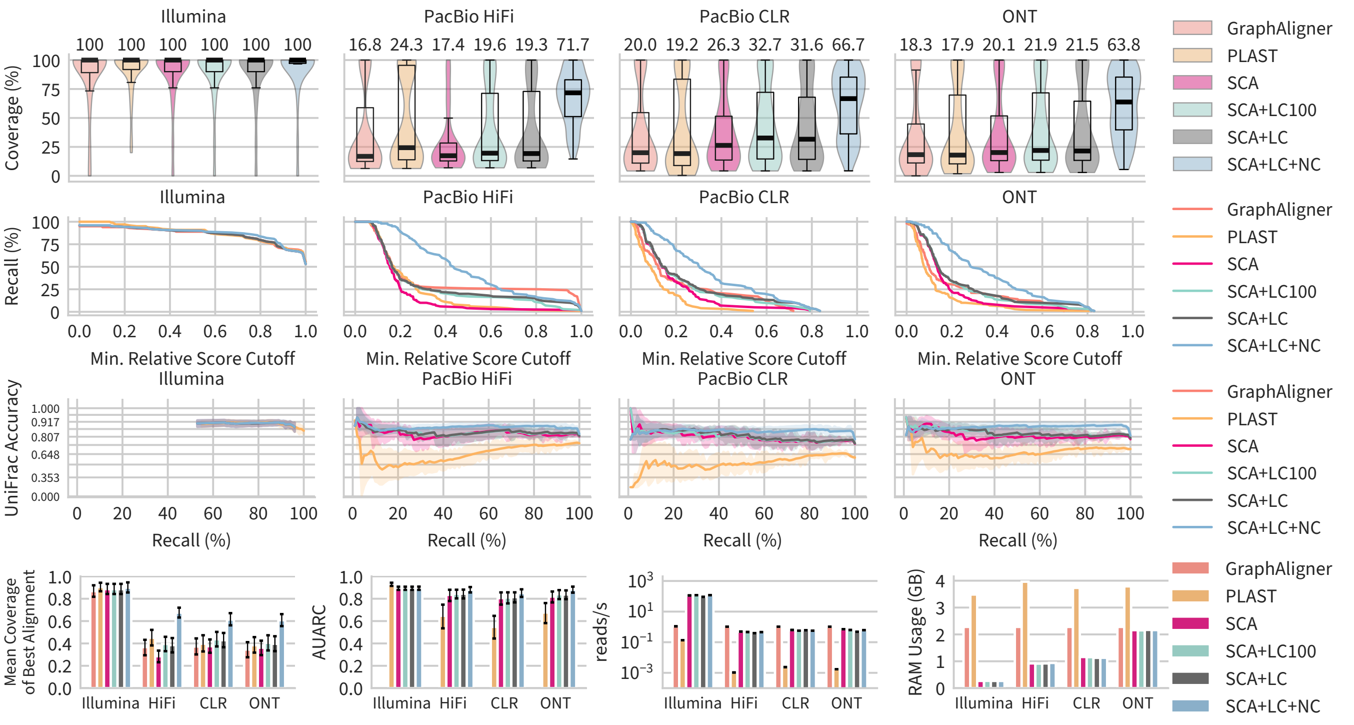

Fig. 3: **Extensions of Main Figures 4, 6, 5, and 7 including the additional baseline** `SCA+LC100`**.**

4. Supplementary Tables

Table 3. Evaluation measures for the alignments produced by each tool. Values between brackets represent 95% confidence intervals from 1000 bootstrap samples.

Platform	Measure / Method	AUARC	Coverage of Best Alignment	RAM Usage (GB)	reads/s
Illumina	GraphAligner	N/A	0.868 [0.817,0.920]	2.281	1.090 [1.090,1.090]
	PLAST	0.930 [0.915,0.943]	0.904 [0.867,0.943]	3.486	0.135 [0.135,0.135]
	SCA	0.895 [0.880,0.908]	0.886 [0.842,0.933]	0.270	113.643 [113.643,113.643]
	SCA+LC100	0.895 [0.880,0.908]	0.886 [0.842,0.933]	0.272	118.749 [118.749,118.749]
	SCA+LC	0.895 [0.880,0.908]	0.886 [0.842,0.933]	0.270	91.447 [91.447,91.447]
	SCA+LC+NC	0.895 [0.880,0.908]	0.899 [0.856,0.946]	0.269	119.478 [119.478,119.478]
PacBio HiFi	GraphAligner	N/A	0.368 [0.294,0.432]	2.281	1.038 [1.038,1.038]
	PLAST	0.648 [0.535,0.747]	0.451 [0.382,0.521]	3.963	0.001 [0.001,0.001]
	SCA	0.835 [0.780,0.880]	0.286 [0.232,0.336]	0.921	0.497 [0.497,0.497]
	SCA+LC100	0.845 [0.803,0.880]	0.395 [0.331,0.459]	0.913	0.464 [0.464,0.464]
	SCA+LC	0.845 [0.803,0.882]	0.385 [0.320,0.448]	0.920	0.393 [0.393,0.393]
	SCA+LC+NC	0.881 [0.856,0.904]	0.676 [0.632,0.720]	0.939	0.459 [0.459,0.459]
PacBio CLR	GraphAligner	N/A	0.373 [0.302,0.445]	2.281	1.039 [1.039,1.039]
	PLAST	0.548 [0.444,0.647]	0.398 [0.328,0.473]	3.733	0.002 [0.002,0.002]
	SCA	0.806 [0.747,0.857]	0.375 [0.316,0.436]	1.161	0.631 [0.631,0.631]
	SCA+LC100	0.811 [0.758,0.857]	0.438 [0.373,0.503]	1.154	0.567 [0.567,0.567]
	SCA+LC	0.815 [0.767,0.857]	0.429 [0.366,0.494]	1.128	0.602 [0.602,0.602]
	SCA+LC+NC	0.852 [0.816,0.884]	0.614 [0.562,0.671]	1.130	0.567 [0.567,0.567]
ONT	GraphAligner	N/A	0.346 [0.276,0.411]	2.281	1.028 [1.028,1.028]
	PLAST	0.677 [0.582,0.761]	0.384 [0.318,0.456]	3.790	0.002 [0.002,0.002]
	SCA	0.820 [0.767,0.865]	0.362 [0.296,0.425]	2.161	0.714 [0.714,0.714]
	SCA+LC100	0.840 [0.797,0.877]	0.406 [0.337,0.473]	2.153	0.629 [0.629,0.629]
	SCA+LC	0.838 [0.797,0.875]	0.397 [0.331,0.464]	2.169	0.480 [0.480,0.480]
	SCA+LC+NC	0.882 [0.854,0.908]	0.608 [0.555,0.662]	2.163	0.618 [0.618,0.618]

Table 4. Breakdown of execution times (in seconds) for each component of the alignment workflow.

		Chaining		Extension		MLATime		Overhead		Time (s)
Platform	Method	mean±std	%	mean±std	%	mean±std	%	sum	%	sum
Illumina	SCA	0.002 ± 0.007	25.342	0.004 ± 0.009	43.071	0.000 ± 0.000	0.000	0.278	31.587	0.880
	SCA+LC100	0.002 ± 0.007	25.650	0.004 ± 0.008	43.462	0.000 ± 0.000	0.119	0.259	30.770	0.842
	SCA+LC	0.003 ± 0.008	22.862	0.005 ± 0.010	42.431	0.000 ± 0.000	0.000	0.380	34.707	1.094
	SCA+LC+NC	0.002 ± 0.007	24.732	0.004 ± 0.008	44.804	0.000 ± 0.000	0.119	0.254	30.345	0.837
PacBio HiFi	SCA	1.034 ± 3.260	51.367	0.825 ± 1.267	41.021	0.000 ± 0.000	0.000	15.319	7.613	201.226
	SCA+LC100	1.022 ± 3.217	47.449	0.852 ± 1.270	39.552	0.125 ± 0.264	5.814	15.471	7.185	215.323
	SCA+LC	1.075 ± 3.349	42.225	1.086 ± 1.555	42.630	0.176 ± 0.361	6.895	21.006	8.249	254.631
	SCA+LC+NC	1.006 ± 3.174	46.134	0.824 ± 1.231	37.789	0.203 ± 0.425	9.317	14.739	6.760	218.045
PacBio CLR	SCA	0.183 ± 0.381	11.571	1.331 ± 2.589	83.999	0.000 ± 0.000	0.000	7.022	4.431	158.497
	SCA+LC100	0.193 ± 0.403	10.948	1.432 ± 2.651	81.129	0.058 ± 0.180	3.261	8.227	4.662	176.482
	SCA+LC	0.183 ± 0.380	11.015	1.338 ± 2.578	80.595	0.067 ± 0.207	4.060	7.187	4.330	165.986
	SCA+LC+NC	0.196 ± 0.410	11.135	1.411 ± 2.648	80.000	0.078 ± 0.227	4.445	7.794	4.420	176.329
ONT	SCA	0.289 ± 0.839	20.616	1.033 ± 1.670	73.745	0.000 ± 0.000	0.000	7.900	5.639	140.087
	SCA+LC100	0.284 ± 0.825	17.877	1.033 ± 1.661	65.009	0.193 ± 1.362	12.162	7.869	4.952	158.897
	SCA+LC	0.329 ± 0.938	15.780	1.417 ± 2.264	67.976	0.221 ± 1.510	10.626	11.711	5.618	208.450
	SCA+LC+NC	0.282 ± 0.824	17.428	0.998 ± 1.610	61.674	0.265 ± 1.860	16.371	7.325	4.527	161.794

References

C. Jain, D. Gibney, and S. V. Thankachan. Algorithms for colinear chaining with overlaps and gap costs. *Journal of Computational Biology*, 29(11):1237–1251, 2022. doi: 10.1089/cmb.2022.0266.
